# Radical versus supraomohyoid neck dissection in the treatment of squamous cell carcinoma of the inferior level of the mouth

**DOI:** 10.1016/S1808-8694(15)30124-5

**Published:** 2015-10-19

**Authors:** Abrão Rapoport, Daniel Kanabben Ortellado, Ali Amar, Carlos Neutzling Lehn, Rogério Aparecido Dedivitis, Ricardo Salinas Perez, Helen Mara Rodrigues

**Affiliations:** a Professor in the USP Medical School. Technical Director of the Health Dept. - Heliopolis Hospital; b Master in health sciences, HOSPHEL, Heliopolis Hospital, Sao Paulo; c Doctor, Otorhinolaryngology Department, UNIFESP - Sao Paulo Federal University, Physician and surgeon of the Otorhinolaryngology and Head & Neck Department, HOSPHEL - Heliopolis Hospital, Sao Paulo; d Doctor, Otorhinolaryngology Department, UNIFESP - Sao Paulo Federal University. Head of the Otorhinolaryngology and Head & Neck Surgery, HOSPHEL - Heliopolis Hospital, Sao Paulo; e Doctor, Otorhinolaryngology Department, UNIFESP - Sao Paulo Federal University, Permanent faculty of the graduation course in medicine (Health Sciences), HOSPHEL, Heliopolis Hospital, Sao Paulo; f Master’s degree student - Medicine (Health Sciences), HOSPHEL - Heliopolis Hospital, Sao Paulo; g Master’s degree student - Medicine (Health Sciences), HOSPHEL - Heliopolis Hospital, Sao Paulo

**Keywords:** mouth, neck dissection, radical, supraomohyoid

## Abstract

The therapeutic paradigm for neck metastasis of squamous cell carcinoma (SCC) in the lower level of the mouth has changed due to survival. **Aim:** A comparative study between radical (RND) versus selective neck dissection (SND). **Material and Method:** An analysis of mouth SCC in the lower level of the mouth in 460 files from the Head & Neck and ORL Department of the Heliopolis Hospital, from 1978 to 2002. In the RND the metastathic rate in levels IV and V was assessed; in the SND the presence and site of recurrence was identified. The chi square test with the Yates correction was the chosen statistical method. **Results:** In the RND the metastatic rates were 5.8% (level IV) and 4.6% (level V) for cNO cases, and 9.9% (level IV) and 5.9%(level V) for cN+ cases; for level I only the rates were 11,0% for cNO cases and 5.5% for cN+ cases. In the SND the number of recurrences was 4 (4.1%) in 97 neck dissections (pNO) and 2 (10%) in 20 neck dissections (pN+). There was no advantage in using radiation compared to non-irradiated cases (5.6% and 5.7%). **Conclusion:** The SND may be used for SCC of the lower level of the mouth.

## INTRODUCTION

The presence of lymphatic cervical metastases in patients bearing epidermoid carcinoma (or squamous cell carcinoma) of the lower portion of the mouth predicts an unfavorable prognosis. This explains a constant concern and strategy changes in the treatment of the neck with this malignancy. Originally, radical neck dissection was the rule for therapy.^1^ This approach started to be questioned due to lack of disease control^2^ and increased knowledge about visceral compartments that justified the preservation of non-lymphatic structures of the neck (jugular vein, spinal nerve and the sternocleidomastoid muscle).^3^, ^4^ After the 1970s, new studies revealed that lymphatic metastasizing into various neck regions followed a selective pattern according to the site of the primary tumor^5^, ^6^, ^7^ This made it possible to progressively systematize selective neck dissection, where results were similar those obtained from radical neck dissection, although some of the lymphatic chains were not resected.8 These findings enabled physicians to recommend selective neck dissection with oncological safety. Initially, this procedure was used in necks with no metastatic lymph nodes (cN0) and in those cases with lymph node metastases up to 2 cm with no rupture of the capsule (pN1).^9^, ^10^, ^11^, ^12^, ^13^, ^14^, ^15^, ^16^, ^17^ These studies underlined our proposition to change the extension of neck dissection for the surgical treatment of necks with clinical metastases (cN1) from squamous cell carcinoma in the lower region of the mouth. We based our proposition on the follow-up of patients and the diagnosis of lymph node recurrence in those anatomical regions not included in the selective procedure.

## MATERIAL AND METHOD

A retrospective analysis was made of 460 charts of patients diagnosed with squamous cell carcinoma in the lower region of the mouth. The Research Ethics Committee approved the trial (number 353). Eligibility criteria were as follows: previously untreated patients with squamous cell carcinoma in the lower region of the mouth (tongue, floor of the mouth, retromolar region and the lower gingiva) that underwent radical or selective (supraomohyoid) neck dissection, with a minimum follow-up period of 12 months or until death.18 Patients were classified according to age, sex, site of the primary tumor, and clinical and pathological staging (TNM 2002). The mean age was 54.5 years, the median age was 53 years (Q25-75% = 47 - 62), the minimum age was 22 years and the maximum age was 87years. There were 406 men (88.3%) and 54 women (11.7%), an 8:1 ratio. The tumor sites were the floor of the mouth (180 cases, 39.1%), the tongue (136 cases, 29.6%), the retromolar region (74 cases, 16.1%) and the lower gingiva (70 cases, 15.2%).

Staging of the primary tumor was as follows: 14 cases were T1 (3.0%), 157 cases were T2 (34.1%), 146 cases were T3 (31.8%), 138 cases were T4 (30.0%) and five cases were 5 Tx (1.1%). Clinical staging of the neck was as follows: 227 cases were cN0 (49.3%) and 233 cases were cN+ (50.7%), of which 119 cases were N1 (25.9%), 18 cases were N2a (3.9%), 58 cases were N2b (12.6%), 23 cases were N2c (5.0%), 14 cases were N3 (3.0%) and 1 case was Nx (0.2%) (Table 1). Patients were grouped according to the clinical stage of the disease, as follows: Stage I - 8 cases (1.7%), Stage II - 101 cases (21.9%), Stage III - 152 cases (33.1%) and Stage IV - 196 cases (42.6%). Clinical staging was impossible in three cases (0.7%).

**Table 1:** Clinical T and N staging (cT and cN) of 460 patients with squamous cell carcinoma of the oral cavity.

	cN0	cN1	cN2a	cN2b	cN2c	cN3	cNx	TOTAL%
cT1	8	1	0	2	1	1	1	14(3,0%)
cT2	101	38	2	11	1	4	0	157(34,1%)
cT3	71	41	6	20	5	3	0	146(31,8%)
cT4	45	37	9	25	16	6	0	138(30,0%)
cTx	2	2	1	0	0	0	0	5(1,1%)

TOTAL	227 (49,3%)	119 (25,9%)	18 (3,9%)	58 (12,6%)	23 (5,0%)	14 (3,0%)	1 (0,2%)	460 (100,0%)

T = staging of the primary tumor - TNM. N = staging of lymph node metastasis - TNM

The pN staging was as follows: 214 cases (46.5%) were pN0 and 246 cases (53.5%) were pN+, of which 62 cases (13.5%) were pN1, 7 cases (1.5%) were N2a, 138 cases (30.0%) were pN2b, 23 cases (5.0%) were pN2c, 5 cases (1.1%) were pN3 and 11cases (2.4%) were unclassifiable (pNx) (Table 2).

**Table 2:** Comparative study of cN and pN parameters.

Clinical N stage	Stage N, on pathology							TOTAL
	pN0	pN1	pN2a	pN2b	pN2c	pN3	pNx	
N0	152 (66,9%)	29 (12,8%)	0	41 (18,1%)	1 (0,4%)	0	4 (1,8%)	227 (100%)
N1	41 (34,5%)	19 (15,9%)	2 (1,7%)	48 (40,3%)	4 (3,4%)	0	5 (4,2%)	119 (100%)
N2a	2 (11,1%)	4 (22,2%)	2 (11,1%)	7 (38,9%)	3 (16,7%)	0	0	18 (100%)
N2b	11 (18,9%)	8 (13,8%)	2 (3,5%)	30 (51,7%)	5 (8,6%)	0	2 (3,5%)	58 (100%)
N2c	6 (26,1%)	2 (8,7%)	0	5 (21,7%)	10 (43,5%)	0	0	23 (100%)
N3	2 (14,3%)	0	1 (7,1%)	6 (42,9%)	0	5 (35,7%)	0	14 (100%)
Nx	0	0	0	1 (100%)	0	0	0	01 (100%)

TOTAL	214	62	7	138	23	5	11	460

N = clinical staging of lymph node metastasis - TNM, pN = staging of lymph node metastasis on pathology - TNM

All of the patients underwent elective/therapeutic treatment of the primary tumor and the neck simultaneously. Surgery was unilateral or bilateral radical or selective (supraomohyoid) neck dissection. Local, regional and distance recurrences were assessed, as were second primary tumors and their sites. In regional recurrences, control rates of the primary tumor, the site of recurrence (within or out of the operated area) and the rate of postoperative radiotherapy were evaluated.

Statistics comprised the descriptive method; qualitative variables were evaluated using the chi-square test with the Yates correction. The Statistica 5.0 (Statsoft, USA) software was used.

## RESULTS

There were 445 radical neck dissections, distributed according to metastasizing levels (Table 3). In 273 cases N+ that excluded 2Nx, the cases were distributed according to those levels and TNM staging (Table 4). The distribution of lymph nodes according to the four sites of the lower region of the mouth was sub-classified for the 387 radical neck dissections (Table 5). In supraomohyoid neck dissections (among the total 573), 22 cases (17.1%) presented metastatic lymph node involvement (pN+); 106 cases (82.9%) had no metastases on histopathology (pN0). The rates of local, regional and local-regional recurrence are shown on Table 6 for those 106 pN0 cases (82.9%). There was regional recurrence in 27 cases (6.1%) of 445 neck dissections. Of these 7 cases (4.0%) were pN0 and 20 cases (7.3%) were pN+. In those 7 pN0 cases, 5 cases recurred within the dissection area and 2 recurred in the contralateral side of the neck; these 7 were not irradiated. Of the 20 pN+ cases, 11(2,2%) occurred in the neck, 2 within the dissected area and 9 in the contralateral side of the neck; 4 of these cases were irradiated. Regional recurrence occurred in 9 cases (7.1%) of 128 supraomohyoid neck dissections, of which 6 cases (7.3%) were pN0 and 3 cases were pN+ (6.5%). Recurrence was within the dissected area in 2 of the 6 pN0 cases, and in the contralateral side of the neck in 2 cases; none of these cases were irradiated. In 3 pN+ recurrence cases one occurred within the dissected area, another case was ipsilateral, not in the dissected area (no adjuvant radiotherapy was used in these two cases), and a third case recurred in the contralateral side of the neck (this patient underwent postoperative radiotherapy). In 22 pN+ patients there were level Ia metastases in 5 cases, Ib metastases in 6 cases, IIa metastases in 8 cases and level III metastases in 7 cases. Table 7 shows local (RL), regional (RR) and local-regional (RL+RR) recurrences in this group. There was no statistically significant difference between the absence (pN0) and presence (pN+) of metastases on histology and the diagnosis of isolated regional recurrence (Table 8) as a function of neck dissection. Finally, there was no significance in the incidence of isolated unilateral events in 230 unilateral neck dissections (p=1.0). Radiotherapy was done in 9 cases (5.6%) of recurrence out of 160 neck dissections; 4 cases of recurrence (5.7%) out of 70 were not irradiated.

**Table 3:** Distribution of cervical metastases in 445 radical neck dissections - 172 clinically N0 (cN0) and 273 clinically positive (cN+).

Neck levels	cN0	cN+
Ia	3 (1,7%)	18 (6,6%)
Ib	11 (6,4%)	64 (23,4%)
IIa	35 (20,3%)	122 (44,7%)
IIb	7 (4,1%)	15 (5,5%)
III	18 (10,5%)	57 (20,9%)
IV	10 (5,8%)	27 (9,9%)
V	8 (4,6%)	16 (5,9%)

TOTAL	172	273

cN0 = no clinically palpable metastases, cN+ = clinically palpable metastases

**Table 4:** Distribution of lymph node involvement in different levels of the neck according to clinical staging.

Levels	Staging (number of neck dissections)				
	cN1 - 122	cN2a - 22	cN2b - 67	cN2c - 41	cN3 - 19
Ia	4 (3,3%)	1 (4,5%)	6 (8,9%)	6 (14,6%)	1 (5,3%)
Ib	31 (25,4%)	2 (9,1%)	16 (23,9%)	10 (24,4%)	4 (21,0%)
IIa	51 (41,8%)	15 (68,2%)	33 (49,2%)	15 (36,6%)	7 (36,8%)
IIb	5 (4,1%)	0	7 (10,4%)	0	3 (15,8%)
III	18 (14,7%)	4 (18,2%)	20 (29,8%)	8 (19,5%)	6 (31,6%)
IV	12 (9,8%)	1 (4,5%)	5 (7,5%)	6 (14,6%)	3 (15,8%)
V	4 (3,3%)	2 (9,1%)	6 (8,9%)	2 (4,9%)	2 (10,5%)

cN = clinical staging of lymph node metastases - TNM

**Table 5:** Distribution of lymph node involvement in neck levels and sub-levels in 387 patients undergoing radical neck dissection for tongue, gingiva, floor of the mouth and retromolar tumors, in cN0 and cN+ cases.

	Ia	Ib	IIa	IIb	III	IV	V
RETR N0	0	2	11	1	2	3	1
RETR N+	2	8	17	2	3	0	1
SOAL N0	1	4	9	1	5	1	4
SOAL N+	7	26	38	5	28	12	7
GENG N0	2	4	6	2	2	3	0
GENG N+	5	14	14	0	3	3	1
LÍNG N0	0	1	9	3	9	3	3
LÍNG N+	4	10	44	8	20	11	7

N0 = no clinically palpable metastases, N+ = clinically palpable metastases

RETR = retromolar region, SOAL = floor of the mouth, GENG = gingiva, LING = tongue

**Table 6:** Local recurrence (RL), regional recurrence (RR) and local-regional recurrence (RL + RR) in pN0 patients undergoing supraomohyoid neck dissection.

Neck dissection(n)	RL	RR	RL + RR
ESOH (48)	12 (25,0%)	4 (8,3%)	5 (10,4%)
EC + ESOH (13)	2 (15,4%)	1 (7,7%)	2 (15,4%)
ESOHB (10)	2 (20,0%)	1 (10,0%)	0

ESOH = unilateral supraomohyoid neck dissection; EC + ESOH = radical neck dissection + supraomohyoid neck dissection; ESOHB = bilateral supraomohyoid neck dissection.

**Table 7:** Local recurrence (RL). regional recurrence (RR) and local-regional recurrence (RL + RR) in pN+ patients undergoing supraomohyoid neck dissection

Neck dissection(n)	RL	RR	RL + RR
ESOH (9)	2 (22,2%)	1 (11,1%)	1 (11,1%)
EC + ESOH (25)	6 (24,0%)	4 (16,0%)	1 (4,0%)
ESOHB (6)	2 (33,3%)	0	0

ESOH = unilateral supraomohyoid neck dissection; EC + ESOH = radical neck dissection + supraomohyoid neck dissection; ESOHB = bilateral supraomohyoid neck dissection.

**Table 8:** Recurrence according to the type of neck dissection and the absence (pN0) and presence (pN+) of metastases on histology

Neck dissection/Isolated regional recurrence	Radical	Supraomohyoid	Total	p
pN0	5(157) = 3,1%	4(97) = 4,1%	9/254 = 3,5%	0,96
pN+	11(253) = 4,3%	2(20) = 10,0%	13/273 = 4,7%	0,24

Total	16(40) = 3,9%	6(117) = 5,1%	6/117 = 5,1%	-

## DISCUSSION

One of the methods of assessing the feasibility of therapeutic selective (supraomohyoid) neck dissection is to study the distribution of cN0 and cN+ levels in the 445 radical neck dissections (Table 3). Level IV was seen in 9.9% of cases and level V was seen in 5.9% of cases; Shah found a 3% rate of level IV involvement for N0 cases and 15% for N+ cases. This author also noted that level V was rarely involved and was always accompanied by metastases in other lymph node levels. Based on this analysis three forms or recurrence were defined in or out of the dissected area and unilateral or contralateral. Our results were 3.9% in radical neck dissection and 5.1% in supraomohyoid neck dissection; these results are similar to those reported in the literature^9^, ^12^ in which the respective values are 9.3% and 10.2%. Our analysis of recurrence and the presence of metastases verified on histology, we found 3.5% for pN0, and 4.7% for pN+, while these numbers in the literature are 5% and 10%.^19^

In cases of recurrence, 5,6% of cases underwent radiotherapy and 5,7% of cases had no radiotherapy, which is similar to the literature.^5^, ^14^, ^20^, ^21^, ^22^

We concluded that the choice of supraomohyoid neck dissection for squamous cell carcinoma in the lower region of the mouth is feasible without compromising oncological results.

## CONCLUSION

The choice of selective neck dissection in levels I to IV in cases of squamous cell carcinoma in the lower region of the mouth associated with palpable metastases at level I is feasible without loss of oncological results.
